# Enabling Demand Side Management: Heat Demand Forecasting at City Level

**DOI:** 10.3390/ma12020202

**Published:** 2019-01-09

**Authors:** Petri Hietaharju, Mika Ruusunen, Kauko Leiviskä

**Affiliations:** Control Engineering, Environmental and Chemical Engineering, University of Oulu, P.O. Box 4300, FI-90014 Oulu, Finland; mika.ruusunen@oulu.fi (M.R.); kauko.leiviska@oulu.fi (K.L.)

**Keywords:** district heating, heat demand, prediction, building, parameter estimation, demand response, NARX

## Abstract

Implementation of new energy efficiency measures for the heating and building sectors is of utmost importance. Demand side management offers means to involve individual buildings in the optimization of the heat demand at city level to improve energy efficiency. In this work, two models were applied to forecast the heat demand from individual buildings up to a city-wide area. District heating data at the city level from more than 4000 different buildings was utilized in the validation of the forecast models. Forecast simulations with the applied models and measured data showed that, during the heating season, the relative error of the city level heat demand forecast for 48 h was 4% on average. In individual buildings, the accuracy of the models varied based on the building type and heat demand pattern. The forecasting accuracy, the limited amount of measurement information and the short time required for model calibration enable the models to be applied to the whole building stock. This should enable demand side management and lead to the predictive optimization of heat demand at city level, leading to increased energy efficiency.

## 1. Introduction

In developed countries, buildings account for 20–40% of the total energy consumption and half of this energy is used by heating, ventilation and air conditioning (HVAC) systems [1]. Furthermore, for 15 of the 28 EU countries the annual heat demand in buildings presents the largest energy demand surpassing electricity and cooling demands [2]. Also, most of the heat is still being produced with fossil fuels [3]. In 2012, the Energy Efficiency Directive (EED) [4] established binding measures for EU countries to improve energy efficiency by 20% at EU level by 2020 and in 2016, an update to EED was proposed setting a new 30% energy efficiency target for 2030 [5]. For the above mentioned reasons, the implementation of new energy efficiency measures for the heating and building sectors is of utmost importance. In this article two different modelling approaches for forecasting the heat demand from individual buildings up to city level are presented. It is argued that relatively straightforward methods would be an important asset for improving the optimization of heat demand for large buildings and heating systems, such as district heating networks, enabling peak load cutting and demand side management actions leading to increased energy efficiency.

As advantages of city-level energy forecasts, Tardioli et al. [6] list the identification of demand response actions and peak power demand, for instance. However, they also state that city-level consumption forecasts can be extremely time-consuming if the simulations are done on the single building level, due to data gathering, simulation and monitoring efforts and the estimation of uncertainties. Consequently, forecast models are widely used for individual buildings, but their application at the large scale is lacking [6,7,8]. Others have even stated that it is impossible to model every building separately, one of the main reasons being the lack of real measurement data [9]. However, today many buildings are equipped with smart meters that record heat consumption in the intervals of an hour or less. Furthermore, model predictive control (MPC) has been one of the most studied control strategies for buildings during the last decade, offering an efficient way to perform demand response actions in buildings, but the amount of modelling work required makes implementation expensive [10,11,12]. Ease of modelling would make the forecasting of heat demand and implementation of predictive control strategies at the building and city level more cost-effective.

Many of the previous works have considered only the total heat demand forecast of a district heating system. These have included data-driven methods like support vector machines (SVM) [13,14], artificial neural networks (ANN) [14,15,16,17,18,19,20] and linear regression [14,19,20,21,22]. Other methods have included a grey-box model [23], a physical-based dynamic system model [24] and a simple piecewise linear model [25]. However, all these approaches would not enable demand side management actions as forecasts for individual buildings are not included. Most of the studies that considered heat demand forecast in individual buildings have had only one building for the model development and testing [26,27,28,29,30,31,32,33]. Applying these models to a larger building stock using the same model structure would not necessarily result in the same accuracy. Some studies have also utilized data from simulated buildings [34,35,36]. Then the model performance in real buildings may remain questionable. There are studies that have considered more than one real building. Ma et al. [37] utilized a Gaussian mixture model for heat demand forecast in nine different buildings. Bacher et al. [38] used a grey-box model to forecast heat demand in 16 detached houses. Machine learning techniques were applied by Idowu et al. [39] to forecast heat demand in 10 residential and commercial buildings. Lauster et al. [40] applied a low order thermal network model to simulate the heat load of a research campus with 200 buildings, but considered only the office buildings. The annual energy use of 1.1 million buildings in New York City was predicted using statistical models by Kontokosta and Tull [41]. However, to authors best knowledge, there appears to be no study where hourly heat demand for a large district heating system has been forecast utilizing models for real individual buildings at city level.

In this work, two different modelling approaches are presented to forecast the heat demand at city level. The proposed modelling approaches forecast the heat demand for individual buildings and at city level, enabling demand side management. The goal of the research was to investigate what level of accuracy could be achieved by applying straightforward modelling methods to forecast the heat demand at city level. A low number of estimated parameters reduces the calculation time and easily attainable measurement data facilitates the implementation of the models for thousands of buildings. By applying models with the same model structure, the generalizability of the models could be assessed. Specifically, the novelty of this study is:

- The utilization of easily adaptable models to forecast the heat demand for over 4000 real individual buildings;
- The forecast of the total heat demand for a large district heating system is based on the models of individual buildings;
- The uncertainty of the weather forecast affecting the modelling accuracy is addressed quantitatively.

The measurement data used for the validation of the forecasting methods is presented in the next section. Section 3 describes the modelling and analysis methods considered. After that, Section 4 presents the identification and validation results which are discussed in Section 5. Finally, Section 6 contains conclusions.

## 2. Measurement Data

The measured district heating data for the city of Jyväskylä, Finland, during 2013, was provided by a local heating utility. The data included hourly recorded heat demand (*P*) and cubic content (*V*) for 4061 individual buildings. Hourly recorded outdoor temperature (*T_out_*) was also available for the same time period.

Heating season data was used for the identification and validation of the heat demand model. In this study, the heating season included the months from January to April and October to December, which amounted to a total of seven months of data. From this data, ten 48-h periods were randomly sampled for the validation of the heat demand model and one to two weeks of data prior to the validation period were used for identifying the model parameters (Table 1). The reason for using a 48-h prediction horizon for model validation comes from the fact that trading in the Scandinavian electricity market is performed one day in advance, making forecasts for the next day the most important for a combined heat and power (CHP) plant. In addition, the uncertainty of the weather forecast was presumed to increase the unreliability and thus hinder the practicality of longer forecasts.

Based on the heat demand data, the buildings were categorized in eight different groups: (1) schools and kindergartens, (2) apartment buildings, (3) apartment buildings with offices, (4) row houses, (5) detached houses, (6) commercial, office and public buildings, (7) manufacturing and warehouses and (8) other buildings. The building groups are presented in Table 2 together with the number of buildings in the group and their contribution to the total heat demand for heating season 2013. In addition, three main heat demand patterns could be identified from the heat demand data of buildings. In some buildings, the heat demand correlated highly with the outdoor temperature, with some buildings showing very smooth heat demand curve while others had more variation. In other buildings, a clear daily pattern could be identified where the heat demand was high at the day time and low at the night time. Then in some buildings the heat demand correlated weakly with the outdoor temperature and there were no identifiable daily patterns.

## 3. Modelling and Analysis Methods

Two different modelling methods were utilized in forecasting the heat demand. The first model (model A) is a dynamic model that builds on the idea of Newton’s cooling law which states that the rate of heat loss of an object is proportional to the temperature difference between the object and its surroundings. A similar approach has been successfully used for predicting the indoor temperature in buildings [42]. As ANNs are one of the state-of-the-art methods for forecasting the building heat demand, a non-linear autoregressive neural network model with external inputs (NARX) was chosen as the second model (model B).

### 3.1. Model A

The applied dynamic heat demand model predicts the future heat demand based on the past heat demand and the heat loss caused by the temperature difference between the indoor and outdoor temperatures. The model is presented in Equation (1) and it forecasts the heat demand for individual buildings:

(1) $$\hat{P}\left(t\right)=a\left[P\left(t-1\right)-U\left(T_{in}\left(t\right)-T_{out}\left(t\right)\right)\right]+b,$$

where $\hat{P}\left(t\right)$ is the forecast heat demand at time *t*, *P*(*t* − 1) is the measured heat demand at time *t* − 1, *T_in_*(*t*) is the indoor temperature at time *t*, *T_out_*(*t*) is the outdoor temperature at time *t*, *a* and *b* are estimated model parameters and *U* (kW/K) is a building-specific physical heat loss coefficient. It is assumed here that the indoor temperature (*T_in_*) is a constant value at +21 °C. This introduces error to the output of the dynamic model as, in reality, the indoor temperature varies by some degrees inside buildings. Therefore, the effect of this variation and other social effects are taken into account with a residual model.

The cyclic heating power variation *w* is calculated with a non-parametric residual model for each hour *i* of the day by subtracting the output of the dynamic model (Equation (1)) from the measured heat demand during the hours *i* and then computing the average:

(2) $$w\left(i\right)=\frac{\sum_{k=1}^{N}\left(P_{k}\left(i\right)-{\hat{P}}_{k}\left(i\right)\right)}{N_{i}},$$

where *i* = 0, 1, …, 23, *P_k_*(*i*) and ${\hat{P}}_{k}\left(i\right)$ are the measured and modelled heat demand at hour *i* respectively and *N* is the number of data points from hour *i*. Different heating patterns at weekends are taken into account by calculating the residual model for weekdays using data from weekdays and for the weekends using data from weekends.

When forecasting heat demand, the output of the non-parametric residual model (Equation (2)) is added to the output of the dynamic model (Equation (1)). The total heat demand for multiple buildings at time *t* can then be calculated using Equation (3):

(3) $${\hat{P}}_{tot}\left(t\right)=\sum_{k=1}^{N}\left({\hat{P}}_{k}\left(t\right)+w_{k}\left(t\right)\right),$$

where ${\hat{P}}_{tot}\left(t\right)$ is the forecast total heat demand at time *t*, ${\hat{P}}_{k}\left(t\right)$ is the forecast heat demand for building *k* at time *t* with the dynamic model, *w_k_*(*t*) is the cyclic heating power variation for building *k* at time *t*, and *N* is the total number of buildings. When forecasting multiple hours ahead, previous results for the heat demand forecast are used as inputs to the model.

**Figure 1:**
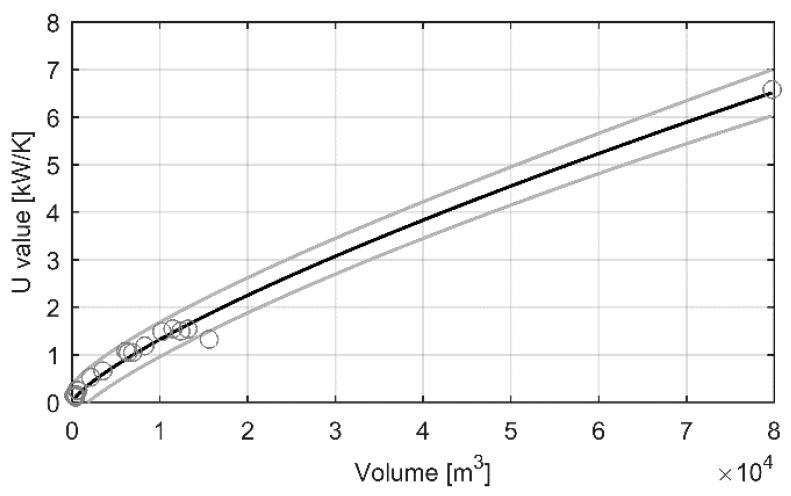
Plot for volume vs. *U* value. Circles are the calculated *U* values, black line is the power function *U* = 0.001119 × *V*^0.7681^ and grey lines are the 95% confidence bounds.

In previous studies [42,43], the physical constants of a similar dynamic model were calculated using area information on the buildings. However, information about the floor, wall and window area of a building is not always easily available and in the case of hundreds or thousands of buildings it could be laborious to collect. Nevertheless, the model has to be applicable to buildings without the aforementioned information in order to achieve wide implementation. In this work, the physical constant *U* was estimated using only the volume of the building. For this, the known calculated values of *U* were plotted against the volume of the buildings and a power function of form *f*(*x*) = *d* × *x^p^* was fitted to the data. The plot is presented in Figure 1 and the resulting equation is *U* = 0.001119 × *V*^0.7681^, where *V* (m^3^) is the volume of the building.

The 95% confidence bounds for the *U* value are also presented in Figure 1. As the real value of the constant *U* for a building is unknown, it could be any value between the confidence bounds, having higher probability of being closer to the fitted line in Figure 1. Therefore, in the validation of the modelling method, the *U* value for a building was randomly chosen from the normally distributed values between the confidence bounds based on the volume of the building.

### 3.2. Model B

**Figure 2:**
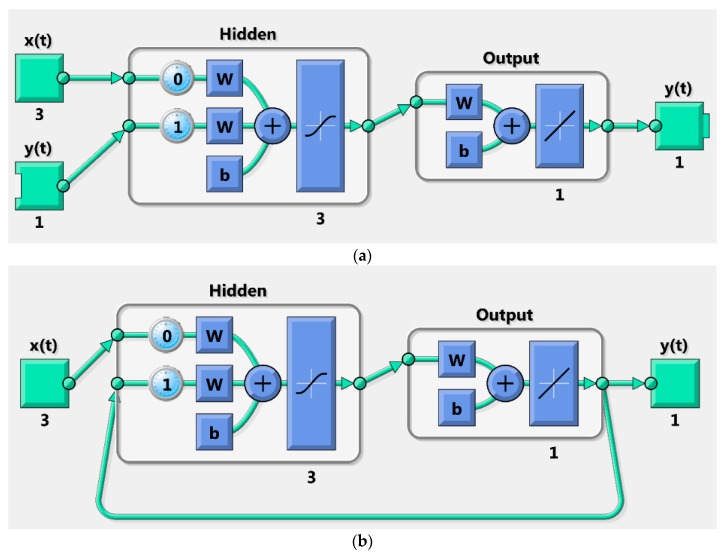
Structure of the neural network model with external inputs (NARX) network where x is the external input, y is the output, w is the weight and b is the bias in: (**a**) Open loop; (**b**) Closed loop.

The applied NARX model forecasts the heat demand based on the previous heat demand and the outdoor temperature, the hour of the day and index for the weekends:

(4) y(t)=f(y(t−1),x(t)),

where *y*(*t*) and *y*(*t* − 1) are the heat demands at time *t* and *t* − 1 respectively and *x*(*t*) consists of the outdoor temperature, the hour of the day (0, 1, …, 23) and index for the weekends (0 or 1) at time *t*. Nonlinear function *f*(∙) is a multilayer perceptron network. One hidden layer was used with three neurons. Tan-sigmoid transfer function was applied in the hidden layer. Figure 2 shows the structure of the NARX network.

### 3.3. Model Performance Analysis

The mean squared error (MSE), root mean squared error (RMSE), absolute percentage error (APE), mean absolute percentage error (MAPE) and Pearson correlation coefficient (r) were applied to evaluate the performance of the forecast models. MSE, RMSE, APE and MAPE are measurements of the difference between the forecast and the real measurements. APE and MAPE present the relative error and are reported in percentages. The Pearson correlation coefficient (r) measures the linear dependence between the forecast and the real measurements. MSE, RMSE, APE, MAPE and r are calculated by Equations (5)–(9) as follows:

(5) $$\mathrm{MSE}=\frac{1}{N}\sum_{t=1}^{N}{\left({\hat{y}}_{t}-y_{t}\right)}^{2}$$

(6) $$\mathrm{RMSE}=\sqrt{\frac{1}{N}\sum_{t=1}^{N}{\left({\hat{y}}_{t}-y_{t}\right)}^{2}},$$

(7) $$\mathrm{APE}=\left(\frac{\left|{\hat{y}}_{t}-y_{t}\right|}{y_{t}}\right)\cdot 100\%,$$

(8) $$\mathrm{MAPE}=\left[\frac{1}{N}\sum_{t=1}^{N}\left(\frac{\left|{\hat{y}}_{t}-y_{t}\right|}{y_{t}}\right)\right]\cdot 100\%,$$

(9) $$r=\frac{N\left(\sum_{t=1}^{N}y_{t}\cdot {\hat{y}}_{t}\right)-\left(\sum_{t=1}^{N}y_{t}\right)\cdot \left(\sum_{t=1}^{N}{\hat{y}}_{t}\right)}{\sqrt{\left[N\sum_{t=1}^{N}y_{t}^{2}-{\left(\sum_{t=1}^{N}y_{t}\right)}^{2}\right]\cdot \left[N\sum_{t=1}^{N}{\hat{y}}_{t}^{2}-{\left(\sum_{t=1}^{N}{\hat{y}}_{t}\right)}^{2}\right]}},$$

where *N* is the number of data points and *ŷ_t_* and *y_t_* are the predicted and the measured values of variable *y* at time *t*, respectively.

In addition, residual analysis was performed to check the distribution of the modelling errors. All the data analysis and modelling were performed in the MATLAB^®^ environment (version R2017a).

## 4. Identification and Validation of the Heat Demand Models

**Figure 3:**
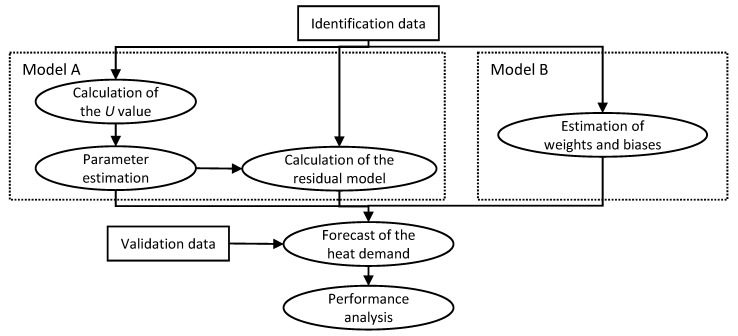
The identification and validation procedure for the heat demand forecast models.

The 10 identification and validation data sets (see Table 1) were used to assess the forecasting performance of the heat demand models. Measured outdoor temperature data was used for identification and validation. Figure 3 describes the applied identification and validation procedure. Data prior the validation periods were used as identification data for training and parameter estimation for models A and B. The model parameters *a* and *b* of the model A (Equation (1)) were estimated by minimizing the RMSE between the actual measured heat demand and the model output utilizing the *pattern search* algorithm in MATLAB^®^. Then the residual model (Equation (2)) was calculated. Different values of the constant *U*, estimated as described in Section 3.1, were used for each data set in each building. Model B was trained in an open loop (Figure 2a) and Bayesian regulation was used as a training function minimizing the MSE. Two-day (48 h) validation data sets were then used to estimate the forecasting performance of the models. Validation of the model B was done in a closed loop (Figure 2b).

Identification and validation were performed on a standard PC with an Intel^®^ Core i7 processor with 3.4 GHz speed, and 8 GB of RAM. Local parallel computing in MATLAB^®^ was utilized. For model A, the calculation time for parameter estimation and validation of 4061 buildings with one data set was approximately eight minutes. The calculation time for model B was approximately 10 minutes. As hourly data is used, and considering the calculation time, it is completely reasonable that the parameters of both models could be updated in a real-world application hourly to ensure the accuracy of the heat demand forecast.

### 4.1. Total Heat Demand Forecast

To identify the amount of historical data required for forecasting the heat demand, the average MAPE, r and RMSE were calculated for ten validation data sets (Table 1) using two weeks and one week of data for parameter estimation and training. For model A, the results showed that one week of data for parameter estimation and for the calculation of the residual model produced the best forecasting results. Furthermore, one week of data ensures that a weekend is also included among the identification data. For model B, two weeks of data for training produced better results compared with one week of data. A different number of neurons in the hidden layer were also tested and three neurons produced the best overall results.

Identification and validation results for the total heat demand forecast of 4061 buildings using one week of data for parameter estimation for model A and two weeks of training data for model B are presented in Table 3 for each data set. Both models show very similar results. MAPE was under 10% for both models with all the validation data sets, with the average MAPE being about 4%. The standard deviations of the average MAPE and RMSE were slightly lower for model B. For both models, r was over 0.90 with all validation data sets.

**Figure 4:**
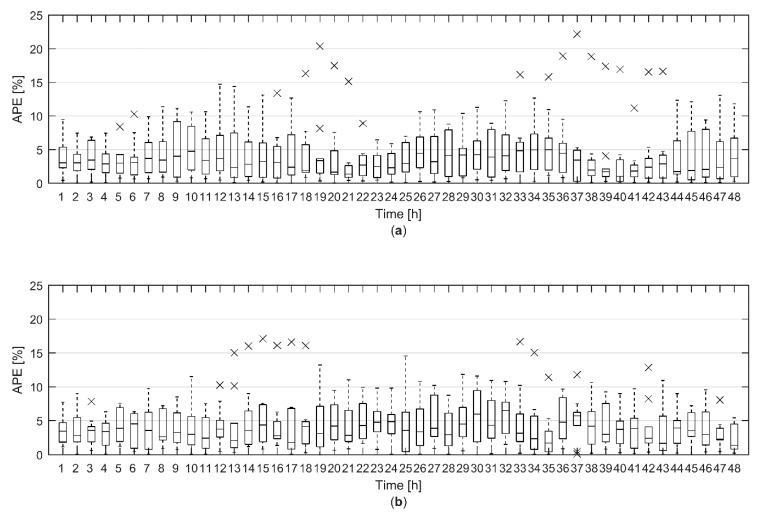
A box plot of the hourly absolute percentage error (APE) for the total heat demand forecast during the 48-h prediction horizon with ten validation data sets for (**a**) model A and (**b**) model B. For each box, the edges are the 25th and 75th percentiles, the central line marks the median, the dashed lines extend to the most extreme data points not considered outliers and the crosses mark the outliers.

The hourly modelling error of the total heat demand forecast during the 48-h prediction horizon with ten validation data sets was further analysed utilizing a box plot, shown in Figure 4. For model A, the median of hourly APE was constantly below 5% for the whole 48-h prediction horizon. For model B, the median of hourly APE was mostly below 5%. There were 23 and 18 outliers for model A and B respectively. These were significantly larger error values than most of the other errors at certain hour. Most of the outliers (15 out of 23) for model A could be identified to originate from validation data set 4, which came from late April. There, the model overestimated the heat demand in the daytime. For model B, the outliers where more evenly distributed. The largest relative modelling errors for model A and B were 22.17% and 17.11% respectively. However, the relative error was under 5% for 79% and 75% of the hours for model A and B respectively. Overall, both models showed very good prediction accuracy for the total heat demand forecast based on the heat demand forecast of 4061 individual buildings.

**Figure 5:**
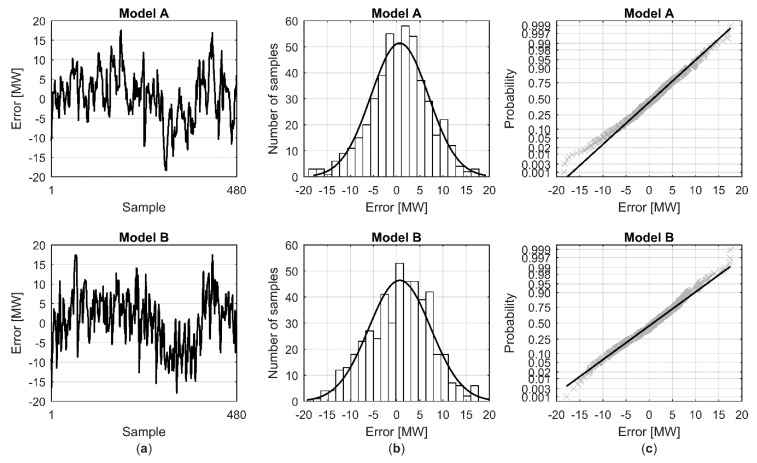
Distribution of the hourly modelling error for the 48-h total heat demand forecast with all ten validation data sets: (**a**) modelling error (model output-measured); (**b**) histogram of the modelling error (white bars) with normal distribution fit (black line); (**c**) normal probability plot for modelling errors.

Distribution of the modelling errors of the total heat demand forecast for all 10 validation data sets (Figure 5a) was analysed by utilizing a histogram together with a normal distribution fit (Figure 5b) and normal probability plot (Figure 5c). The error analysis showed that for both models the modelling errors were almost similar to normal distribution (Figure 5b,c). However, for model A there were some extreme negative residuals that can be seen to form a tail in the normal distribution plot (Figure 5c). Further investigation revealed that these originated from validation data set 7, where the model underestimated the heat demand during one weekend night.

### 4.2. Heat Demand Forecast for Individual Buildings

The applied models forecast the heat demand for all 4061 individual buildings in the study. This is important, because it enables demand side management actions. Therefore, also the forecasts for each individual building have to be accurate, together with the total heat demand forecast.

The average MAPE and r for the 48-h heat demand forecast for individual buildings in different building groups are presented in Table 4. For both models, the performance in individual buildings was significantly worse when compared with the total heat demand forecast (Table 3).

The performance in detached houses is significantly worse than in other groups. This indicates that one explaining factor for the decreased overall performance in individual buildings is the fact that almost half of the studied building stock consists of detached houses. Their heat consumption is low and this produces high MAPE values even though the actual difference between the forecast and measured values is small. Moreover, the more sporadic heat demand patterns of the detached houses, mainly due to hot water consumption, reduce the value of r which is clearly smaller for detached houses compared with other building groups. However, in this study the mean normalized RMSE (RMSE divided by the average heat demand during the forecast period) for the 48-h forecast in 1994 detached houses was 0.32 for both models, showing similar forecasting performance as other forecasting models reported in the literature. Normalized RMSE values from 0.10 to 0.55 were reported in [38] and another study [39] reported values from 0.06 to 0.30. This shows that the forecasting results of the applied models in detached houses are similar to the results of other modelling methods. Furthermore, it should be noted that the accuracy of the total heat demand forecast remains high despite the lower forecasting performance in detached houses due to their low contribution to the total heat demand (about 5%). Nevertheless, in relation to the application of the heat demand forecast in demand side management, the most interesting building groups are apartment buildings, schools and commercial, public and office buildings, namely large buildings whose heat demand is considerably higher than that of detached houses. Also, these are the type of buildings where demand side management would most likely be applied. Therefore, it is important that the forecast model performs well in these buildings.

From the data for the city of Jyväskylä, 1224 apartment buildings, schools and commercial, public and office buildings with an hourly median heat demand of 10 kW or more could be identified. These accounted for 70% of the total heat demand of all 4061 buildings in the study. Average MAPE and r in these buildings were 11.05% and 0.72 for model A and 12.44% and 0.68 for model B, respectively. The forecasting performance for these buildings with 10 validation data sets is also presented in Table 5. For model A, over half of the buildings had the average MAPE lower than 15% and the average r greater than 0.7. Here, model A outperforms model B.

**Figure 6:**
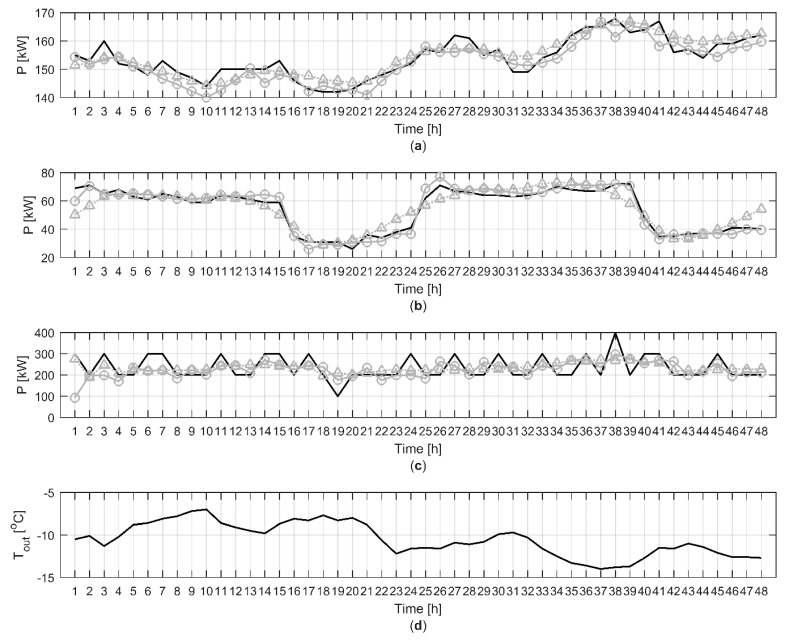
Predicted and measured heat demand in three different buildings for 48 h with validation data set 1: (**a**) apartment building where the heat demand is highly correlated with the outdoor temperature; (**b**) commercial, public or office building where a clear daily pattern in heat demand can be identified; (**c**) apartment building where the heat demand is weakly correlated with the outdoor temperature; (**d**) measured outdoor temperature. The black lines are the measured heat demands and outdoor temperature, the grey lines with circles are the predicted heat demand with model A and the grey dotted lines with triangles are the predicted heat demand with model B.

Further analysis showed that in buildings where the heat demand was highly correlated with the outdoor temperature, the modelling results were generally very good as seen in Figure 6a where MAPE and r were 1.81% and 0.90 for model A and 1.71% and 0.90 for model B, respectively. The modelling results were also generally good in buildings where a clear daily pattern in the heat demand could be identified as in Figure 6b. MAPE and r were 5.25% and 0.98 for model A and 9.34% and 0.90 for model B, respectively. However, in some buildings the heat demand was not following the outdoor temperature very closely as seen in Figure 6c. In these buildings, the model performance was generally poor in terms of MAPE and r which in this case were 17.94% and 0.33 for model A and 18.81% and 0.42 for model B, respectively.

### 4.3. Effect of the Uncertainty in the Weather Forecast on the Heat Demand Forecast

All the previous forecasting results presented were achieved using the measured outdoor temperature. However in a real application, in addition to the forecasting error of the heat demand model itself, the outdoor temperature forecast is a significant source of uncertainty and should be addressed when developing predictive models [44]. Although weather forecasts are quite accurate today, there is always some error in the forecast and it is important to take this into consideration when assessing the performance of a heat demand model [45]. In this work, no past outdoor temperature forecasts were available. Therefore, the inaccuracy of the weather forecast was simulated by introducing disturbance in the measured outdoor temperature based on Wiener process as presented in [46]. The mean value for the outdoor temperature forecast error was assumed to be 0. After *N* hours, the error can be calculated as $\sigma \sqrt{N}$, where *σ* is the hourly standard error. In [46], the hourly standard error for a 5-day outdoor temperature forecast was estimated to be 0.3414 °C based on the forecasts of Finnish Meteorological Institute [47].

**Figure 7:**
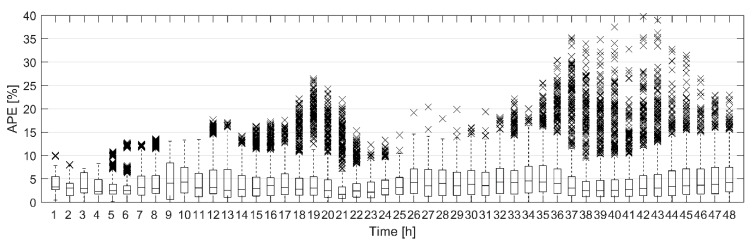
A box plot of the hourly APE for the total heat demand forecast during the 48-h prediction horizon with 10 validation data sets and simulated outdoor temperature forecasts for model A. For each box, the edges are the 25th and 75th percentiles, the central line marks the median, the dashed lines extend to the most extreme data points not considered outliers and the crosses mark the outliers.

To study the effect of the uncertainty of the weather forecast on the total heat demand forecast, the simulated outdoor temperature forecasts for 10 validation data sets were generated as described above. As the outdoor temperature forecast was simulated based on a random process, 100 simulated outdoor temperature forecasts were generated for each validation data set. The total heat demand was then forecast in each validation data set using the simulated outdoor temperature forecasts. Model A was used to make the heat demand forecasts. The modelling errors for the total heat demand forecast during the 48-h prediction horizons with ten validation data sets and simulated outdoor temperature forecasts are presented in Figure 7 as a box plot.

Figure 7 can be compared with Figure 4a where the hourly APE for the heat demand forecast with measured outdoor temperature was presented. It is clear that the uncertainty in the weather forecast increases the modelling error of the heat demand forecast. Although the median hourly APE is still below 5% for the whole 48-h period, the difference between the median APE with simulated outdoor temperature forecasts and the median APE with measured outdoor temperature increases as the prediction horizon gets longer. Also, the dashed lines, which extend to the most extreme values not considered outliers, are much longer in Figure 7 than in Figure 4a. There are many more outliers as can be expected when 100 different simulated outdoor temperature forecasts were used. Still most of the outliers came from the same data sets as in Figure 4a. However, for longer prediction horizons outliers from other data sets were also beginning to show.

Although the forecasting accuracy of the models is good, the weather forecast introduces uncertainty to the forecast of heat demand even for 48-h forecasts. Therefore, it is very important to take this uncontrollable element into account when forecasting heat demand. It is also worth noting that, in this work, the same measured outdoor temperature was used for all the buildings rather than the local outdoor temperature at the exact site of the building. When the heat demand for individual buildings is forecasted, some error is introduced when using the same outdoor temperature for each building as it is quite possible that there are deviations of several degrees in the outdoor temperature between different parts of the city area [48].

## 5. Discussion

Both applied models performed very good at forecasting the total heat consumption at city level. Model A produced slightly smaller MAPE, r and RMSE values, but the standard deviation of these were slightly smaller for model B. In individual buildings, model A performed better than model B. Both models had similar input variables but the amount of estimated parameters were different as seen in Table 6 where a summary of the inputs, outputs, constants and the number of estimated parameters used in the models is presented. Model A has only two free parameters that need to be estimated for each building and this resulted in 20% less time needed for the parameter estimation compared with model B. Also, for model A, only one week of data was needed for the parameter estimation.

The number of external model variables was intentionally kept low to avoid a large number of estimated model parameters and to keep the data needed easily attainable. Therefore, only the outdoor temperature was included from among all the possible weather variables. This is justified by the fact that the heat demand is highly correlated with the outdoor temperature [49] and the difference between indoor and outdoor temperature directly affects the heat loss through the building envelope. The effects of solar irradiation and wind speed are more building specific as Kapetanakis et al. [49] found using a correlation analysis. Nevertheless, in future work, especially for the NARX model (model B), different weather variables could be included for different buildings in order to see if that improves the prediction accuracy. However, then the increased time to find the proper variables and model structure should be also taken into account. Furthermore, these additional weather variables would need to be forecasted and the effect of this on the forecasting performance of the model should then be investigated.

The forecast models presented in this work can be used for the predictive optimization of heat demand. Models are the basis for any MPC and the models presented here are accurate and straightforward to implement. In a daily operation of a district heating system, the models could provide predictive information on the heat demand of the system and individual buildings. This information could then be used to optimize the heat demand for example in order to cut peak loads by utilizing the thermal mass of buildings by applying easily adaptable indoor temperature models [42,43].

The results of this work showed that relatively straightforward modelling methods can produce good results in forecasting the heat demand at city level and in individual buildings. It is hoped that this work can encourage other researchers to apply their models to a larger number of buildings especially if they are to be used for demand side management. As this work demonstrated well, the model performance varied largely between buildings. If only one building would have been chosen, the applied modelling methods could have produced either great or very poor prediction results.

## 6. Conclusions

Two different modelling approaches were applied to forecast the heat demand for individual buildings and at city level. The goal was to study what level of accuracy could be achieved if relatively straightforward modelling methods were utilized. Real measurement data from a large district heating system with more than 4000 buildings was utilized in the validation of the modelling approaches. Forecast simulations with the measured data showed that for a 48-h city level heat demand forecast the MAPE for both models was 4% indicating good modelling performance. However, the weather forecast increases the uncertainty of the heat demand forecast and should be taken into account when making heat demand forecasts. The modelling accuracy of the heat demand forecast for individual buildings was found to be good for large buildings with high correlation between the heating power and outdoor temperature or with a distinctive daily heat demand pattern. For over half of the large buildings, the MAPE was lower than 15% and the r greater than 0.7. In conclusion, the forecasting accuracy and the small amount of information and time needed for the modelling enable the application of the modelling approaches to the whole building stock. This enables the predictive optimization of heat demand at city level, leading to enhanced energy efficiency.

## Figures and Tables

**Table 1:** Data sets for the heat demand forecast with the range of measurement data presented for both the identification data and the validation data.

Data Set	Month ^1^	Range of Measurement Data							
		Identification ^2^				Validation			
		*T_out_* [°C]		Total *P* [MW]		*T_out_* [°C]		Total *P* [MW]	
		Min	Max	Min	Max	Min	Max	Min	Max
1	January	−26.7	−1.0	121.9	298.8	−14.0	−7.0	163.3	222.2
2	April (w)	−18.4	+3.7	97.6	243.6	−7.7	+6.4	86.7	148.1
3	December (w)	−12.3	+5.7	83.5	207.7	+0.8	+4.2	90.0	131.5
4	April (w)	−10.4	+10.7	60.8	180.9	−2.1	+11.0	54.7	115.3
5	April	−18.4	+3.7	97.6	243.6	−9.4	+6.4	86.7	173.8
6	January	−26.7	−1.0	121.9	298.8	−14.0	−8.0	163.3	229.8
7	January (w)	−26.7	−1.0	121.9	298.8	−15.6	−5.0	166.1	239.2
8	October	−5.8	+16.0	45.5	160.3	−1.1	+8.8	84.6	149.2
9	February	−13.8	−0.4	121.3	209.3	−4.3	+5.2	105.1	152.5
10	February (w)	−13.8	+5.2	105.1	209.3	−14.9	+3.9	108.3	194.4

^1^ Month from which 48-h validation data was taken. Periods with (w) included data from weekends. ^2^ The range is presented for identification data two weeks prior to the validation data.

**Table 2:** Different building groups and their number and contribution to the total heat demand for heating season 2013.

Building Group	Number of Buildings	Contribution to Total Heat Demand [%]
Schools and kindergartens	81	9.29
Apartment buildings	923	39.78
Apartment buildings with offices	77	2.81
Row houses	452	7.58
Detached houses	1994	6.18
Commercial, office and public buildings	232	19.16
Manufacturing and warehouses	195	12.90
Others	107	2.30

**Table 3:** Mean absolute percentage error (MAPE), r and root mean squared error (RMSE) for the total heat demand forecast of 4061 buildings with 10 data sets (Table 1). The length of identification data was one week for model A and two weeks for model B.

Data Set	Identification						Validation					
	MAPE [%]		r		RMSE [MW]		MAPE [%]		r		RMSE [MW]	
	A	B	A	B	A	B	A	B	A	B	A	B
**1**	1.58	1.76	0.99	0.99	4.06	4.03	1.29	2.03	0.98	0.96	3.21	5.18
**2**	2.00	2.59	0.99	0.98	3.26	4.68	4.64	6.54	0.98	0.94	5.71	8.69
**3**	1.12	2.16	1.00	0.99	1.94	3.79	3.83	3.82	0.96	0.96	5.23	4.74
**4**	3.13	2.68	0.95	0.99	3.79	3.55	9.43	5.50	0.95	0.97	8.44	5.40
**5**	2.32	2.61	0.98	0.98	4.07	4.95	2.69	4.30	0.99	0.96	3.91	6.47
**6**	1.57	1.72	0.99	0.99	4.12	4.02	1.61	2.15	0.98	0.96	4.20	5.43
**7**	1.76	1.75	0.99	0.99	4.45	3.98	4.33	4.05	0.98	0.98	9.57	8.85
**8**	2.54	3.15	0.97	0.99	4.19	3.64	4.98	5.21	0.96	0.97	6.50	7.92
**9**	1.46	1.74	0.99	0.98	2.81	3.26	4.70	5.88	0.93	0.93	7.20	8.12
**10**	1.68	1.77	0.99	0.99	3.28	3.26	3.27	2.74	0.98	0.99	5.50	4.23
**Average**	1.92	2.19	0.99	0.99	3.60	3.92	4.08	4.22	0.97	0.96	5.95	6.50
**Standard deviation**	0.59	0.52	0.01	0.00	0.77	0.56	2.29	1.57	0.02	0.02	2.02	1.74

**Table 4:** The average MAPE and r for 48-h heat demand forecast with 10 validation data sets in different building groups.

Building Group	Average MAPE (Standard Deviation)		Average r (Standard Deviation)	
	Model A	Model B	Model A	Model B
All buildings	15.17 (8.33)	16.27 (24.61)	0.55 (0.21)	0.54 (0.19)
Schools and kindergartens	11.95 (4.38)	14.17 (8.63)	0.79 (0.16)	0.76 (0.15)
Apartment buildings	10.58 (6.06)	11.48 (6.21)	0.69 (0.13)	0.66 (0.12)
Apartment buildings with offices	13.27 (5.90)	13.92 (6.02)	0.58 (0.23)	0.58 (0.21)
Row houses	13.00 (7.19)	13.43 (7.33)	0.61 (0.17)	0.60 (0.15)
Detached houses	19.93 (6.81)	20.66 (38.00)	0.42 (0.16)	0.43 (0.15)
Commercial, office and public buildings	14.52 (9.64)	18.63 (18.92)	0.77 (0.18)	0.72 (0.17)
Manufacturing and warehouses	17.50 (13.15)	20.25 (14.94)	0.65 (0.23)	0.62 (0.20)
Others	14.79 (7.91)	15.17 (7.66)	0.60 (0.17)	0.59 (0.15)

**Table 5:** The percentage of 1224 large buildings with an average MAPE lower than 30% and an average r greater than 0.5 with ten validation data sets for models A and B (in brackets).

Average r	Average MAPE					
	<30%	<25%	<20%	<15%	<10%	<5%
>0.5	86.1 (84.2)	85.6 (83.7)	84.0 (81.0)	78.2 (74.3)	47.8 (37.7)	0.3 (0.1)
>0.6	77.5 (72.9)	77.0 (72.4)	75.5 (69.9)	70.7 (65.3)	46.5 (36.9)	0.3 (0.1)
>0.7	60.3 (46.3)	59.9 (46.0)	58.6 (44.7)	55.1 (41.1)	39.1 (28.2)	0.3 (0.1)
>0.8	29.8 (16.3)	29.7 (16.2)	28.7 (15.8)	26.6 (14.5)	18.5 (10.1)	0.2 (0.1)
>0.9	6.5 (2.9)	6.5 (2.9)	5.9 (2.8)	5.4 (2.7)	3.3 (2.0)	0.2 (0.1)

**Table 6:** Input variables, outputs, constants, and the number of estimated parameters for model A and B.

Model	Input Variables	Output	Constants	Estimated Parameters	Historical Data
A	*P*, *T_out_*, Time, *V*	Modelled *P*	*T_in_*, *U* value	2	1 week
B	*P*, *T_out_*, Time	Modelled *P*	Lags, Neurons	19	2 weeks
